# Supplementary material for: Are carbon emissions trading and green financial instruments synergistic? -Comprehensive quantitative research based on content analysis
Source: PLoS One. 2024 Mar 7;19(3):e0298601. doi: 10.1371/journal.pone.0298601 (PMC10919601; doi:10.1371/journal.pone.0298601)
Supplement: S1 Data — (ZIP) [file pone.0298601.s001.zip › Data/地级市Z_F工作报告.zip!/║┌┴·╜¡/╞▀╠¿║╙╩╨/└┤╫╘╣1⁄2╓┌║┼í╛┬φ┐╦╩2╛▌═°í┐_2019.docx]

各位代表：

　　现在，我代表市政府，向大会报告政府工作，请予审议，并请政协各位委员提出意见。

　　一、2018年工作回顾

　　日月不肯迟，四时相催迫。过去的一年，我们过得很充实、走得很坚定。在习近平新时代中国特色社会主义思想指引下，在省委、省政府和市委正确领导下，在市人大、市政协监督支持下，我们有效应对经济下行压力，统筹推进稳增长、促改革、调结构、惠民生、防风险各项工作，经济发展平稳向好、转型步伐加快、改革纵深推进、民生持续改善、社会和谐稳定，较好地完成了市十一届人大三次会议确定的各项目标任务。初步统计，全市生产总值增长6.0%左右，规模以上工业增长6.8%，固定资产投资增长7.0%；财政一般公共预算收入实现27.5亿元，增长20.8%，增幅居全省第一位；税费收入突破60亿元，财政总支出突破100亿元，均创历史新高；城镇居民人均可支配收入实现2.5万元，增长6.0%；农村居民人均可支配收入实现1.3万元，增长6.5%，保持与经济增长同步。

　　一年来，我们全面贯彻新发展理念和高质量发展要求，坚定不移调结构转方式，加快新旧动能接续转换，推动产业转型升级，经济发展质量、速度和效益稳步提高。经济结构不断优化，非煤工业增长14.6%，比重提高4个百分点；召开加快民营经济发展千人大会，出台“黄金10条”系列扶持政策，兑现扶持资金6亿元，民营经济比重提高2.7个百分点。产业项目建设成效显著，重点推进千万元以上项目39个，完成投资26亿元，11个项目竣工投产。现代煤化工产业链不断延长，宝泰隆稳定轻烃、勃盛焦化升级改造项目试产运行，成功盘活龙洋、美华、乾丰等焦化企业。新材料产业发展势头强劲，宝泰隆石墨烯通过IGCC认证，石墨烯防腐涂料、高温树脂、万锂泰电池负极材料石墨化项目投产，新材料产业园获批国家石墨及石墨烯特色产业基地。生物和医药产业加速孕育，兴粮30万吨玉米深加工项目建成投产，中瑯生物5万吨衣康酸10万吨葡萄糖酸钠项目完成主体工程建设。制造再制造业提档加速，双叶家具智能制造项目进展顺利，部件加工车间被确定为省第一批数字化（智能）示范车间；升华医疗器械即将通过GMP认证，正式生产；再生资源园区鑫盛矿用产品铸造、欧尔东医用塑料资源化等7个项目投产。现代服务业加快发展，新零售小镇、商贸博览城二期等项目稳步推进，电子商务交易额实现8亿元，全年接待旅游人数超过50万人次。按照“一区多园”模式，统筹园区规划布局，加大基础设施建设力度，重点产业项目全部入园摆放，开发区新入驻企业28户，园区承载集聚能力不断提升。

　　一年来，我们牢牢把握绿特色发展方向，统筹农村一二三产业融合发展，推进农业增效、农民增收，加强生态农业建设和特色品牌培育，现代农业加快发展。农业持续丰产丰收，粮食总产量17.06亿斤。种植业结构不断优化，经济作物播种面积31.1万亩，粮经结构比为88:12。成功处置和防控炭疽、非洲猪瘟疫情，畜牧业稳步发展，规模化养殖场达到142个，天兆牧业百万头生猪项目签约落地。全市龙头企业发展到47个，利健蓝靛果花青素提取、乐宝红豆皂苷饮品、康草堂北药深加工等项目投产运营，农产品加工转化率大幅度提升。推进建设10个农产品质量安全试验示范基地，全市绿色食品认证面积超过40%，有机食品标识认证达到36个、地理标志认证5个。农业科技支撑效果明显，全省农业大数据示范点落户我市，金沙农场被评为省级现代农业产业园。农业经营主体进一步壮大，家庭农场338个、农民合作社992家。成功举办鲁吉黑调味品年会，大米网七台河馆入驻企业42家。龙谷农业科技园、田园音乐葡萄小镇、禹森薰衣草庄园等田园综合体项目进展良好。美丽乡村建设取得新突破，勃利县获全省美丽乡村建设先进县。

　　一年来，我们不断深化改革、扩大开放，以改革促创新，以开放促振兴，经济发展活力有效释放，转型发展内生动力明显增强。深入推进“放管服”改革，审批事项压减三分之一、审批时限压缩三分之二；商事制度改革实现“35证合一”，企业开办时间压缩至8个工作日；落实减税降费政策，环评、安评等14项中介服务费由政府买单，减轻企业税费负担7.85亿元。农村土地承包经营权确权改革完成，集体产权制度改革全面铺开，国有林场改革通过省级验收。扎实推进国有企业改革，七矿公司改革脱困迈出坚实步伐，成功组建市供排水总公司，变“九龙治水”为“一龙统筹”。对外交流合作纵深发展，实现外贸进出口总额685.6万美元，同比增长23%，农交会、哈洽会、进博会等大型展会参展成果丰厚。“江河”合作成效初显，签订合作框架协议16个，签约项目13个，签约额40亿元。开展央地对接活动，云南水务危废中心、辰能生物质发电等项目签约落地。创新发展能力明显增强，建设创业公寓500套、人才公寓100套，引进各类高层次人才67名；与省内外20余所高校和科研院所建立战略合作关系，合作项目120余项；宝泰隆北京石墨烯研究院、鑫科纳米院士工作站、省工研院七台河分院等多家应用技术研发综合体挂牌运行；新认定5户国家高新技术企业、1个省级工程技术研究中心、1个省级重点实验室、18户科技型中小企业。成功举办第二届石墨烯创新创业大赛，6个项目签约；首届中国创翼七台河创业创新大赛，北华电气创新项目获全省一等奖、全国优胜奖。

　　一年来，我们深入推进生态文明建设，坚持规划先行，建管并重，调整优化城市空间布局和生态布局，扎实推进环境综合治理，城乡面貌持续改善。调整完善城市总体规划，启动城市空间规划、综合交通规划编制工作，完成生态保护红线划定工作。新建改造环化路、越秀路、红旗路等15条道路，乌金桥、东进桥、兴学桥3座桥梁，七台河火车站改造完成，北岸新城客运站投入使用，牡佳客专七台河段建设进展顺利，七哈客运实现优化整合，至依兰高铁站客运专线开通，七勃客运公交化运营。实施七矿分散区域集中供热并网工程，新建管网10公里。实施中心区雨水管网续建、东方花园等易涝点整治工程，中心河乡污水处理厂开工建设。深入开展占道经营、违建拆除、油烟净化、道路扬尘等专项整治，拆除违法建筑3900余处，客运总站周边违章建筑全部拆除，光明街大理石商户占道经营全部取缔。辟建文贸夜市，困扰群众多年的户外烧烤污染、环境脏乱差问题得到有效解决。持续推进工业企业超低排放改造，秸秆禁烧保持“零火点”，空气优良天数增长2.3%。深入开展采沉区综合治理，老区道路沉陷断裂和广场破损状况得到历史性改变。全面落实河湖长制，实现四级河长、一河一策全覆盖。强力推进倭肯河流域综合治理，拆违清障1300多处，城区段堤防工程进展顺利，万宝河、新老七台河整治成效显著，实现河畅、水清、岸绿、景美。实施城市绿化美化亮化净化工程，新增绿地面积27.5公顷，国家园林城市顺利通过省级复检验收。建成国家级生态文明乡镇3个、生态村5个，城乡文明建设水平进一步提升。

　　一年来，我们牢固树立以人民为中心的发展思想，尽心竭力强弱项补短板，全心全意办实事惠民生，人民群众获得感、幸福感不断提升。财政民生支出比重达77.6%，30项民生实事扎实推进。脱贫攻坚成效明显，10053户、23440人脱贫，勃利县脱贫摘帽。城市供水工程竣工通水，管网改造全面推进，完成97座水箱水池钛纳米喷涂改造，24小时供水覆盖率由45%提高到80%，成为全国首座自来水直饮全覆盖城市。采煤沉陷区棚户区改造安置1804套，发放货币补偿资金1.65亿元；建设分配廉租房5712套，发放租赁补贴1280万元。七矿物业实行属地管理，311栋弃管楼交由社区代管，完成楼体维修285栋。千方百计促进创业就业，新增就业6659人，城镇登记失业率低于省控指标0.63个百分点。全力办好人民满意教育，新建中小学教学楼2 座，完成4所学校操场改造，改扩建幼儿园9所，中小学校全部实现光纤入校，在全省率先开展三点半课后服务试点工作。新高考准备工作有序开展，高考本科入段率稳步提升。职业教育办学规模不断扩大，护理、汽车、幼教、速滑等品牌学科建设成效明显。特殊教育综合水平居全省前列。公立医院改革深入推进，市人民医院改扩建完成，失能失智老人养护中心投入运行，基本公共卫生服务、重大疾病防控、医疗救治水平明显提升。开展补充医疗保险试点，在全省率先完成城乡医疗基本保险制度整合“六统一”，实行“先诊疗后付费”“两免四减”，贫困人口住院报销比例提高5个百分点。城乡低保标准实现十二连增，社会救助体系不断完善。市文化中心主体工程完工，首届“冰雪嘉年华”系列活动精彩纷呈，成功举办中俄短道速滑邀请赛、“百万青少年上冰雪”等系列活动，向国家和省输送优秀冰雪项目运动员88名。

　　一年来，我们始终把安全稳定作为前提和保障，严责任、强监管、抓整治，创新推进社会综合治理，社会大局和谐稳定。坚决淘汰煤炭落后产能，“真关、真管、真淘汰”，81处矿井关闭到位，退出产能429万吨，兑现奖补资金1.83亿元。创新煤矿监管模式，坚持“制防、技防、人防”综合施策，深入开展综合整治和“百日攻坚”专项行动，地方煤矿连续25个月无死亡事故。健全完善安全风险管控和隐患排查治理双重体系，危险化学品、非煤矿山、道路交通、消防等重点领域、重点场所安全保障切实加强。七台河综合应急救援基地获国家批准支持，列入国家级救援队伍目录。深入开展“法治七台河”建设，“七五”普法顺利通过省级中期检查验收。扎实推进“平安七台河”建设，积极创建全国禁毒示范城市，扫黑除恶专项斗争取得阶段性成果，“9·8”特大地下钱庄系列案件战绩辉煌，全国反洗钱骨干联勤中心落户我市。积极筹措资金化解政府债务，市本级还本付息31.3亿元。严格落实信访工作领导责任制，集中开展“四大攻坚”等专项行动，解决了一大批信访突出问题，越级访量大幅下降，信访形势持续稳定。

　　一年来，我们坚持不懈转作风、优环境，激情工作，苦练内功，用实干、实绩、实效推动各项事业发展，政府自身建设不断加强。认真学习贯彻习近平新时代中国特色社会主义思想和党的十九大精神，坚定正确政治方向，维护权威、捍卫核心、对党忠诚的政治立场更加鲜明。严格执行民主集中制，广泛听取社会各界和人民群众意见建议，严格落实“三重一大”集体决策制度。主动接受人大工作监督、法律监督和政协民主监督，104件人大代表建议和141件政协委员提案全部办结，代表、委员满意率100%。深入推进“互联网+政务服务”，推动政府转职能、强监管、优服务，市本级政府工作部门整合减少5个、新组建4个，由改革前的33个调整为32个，完成政府机构改革阶段性任务。严格落实中央八项规定精神，全面加强政府系统廉政建设，持续推进正风肃纪和反腐败斗争，强化财政资金监管和审计监督，纠正违规问题资金2776万元。

　　各位代表，过去的一年，我们牢固树立“四个意识”，坚决有力地贯彻落实党中央国务院、省委省政府和市委各项决策部署，确保了全市经济社会发展沿着正确的方向行稳致远。我们牢牢把握发展第一要务，坚持走好转型发展“三条根本出路”，大力实施“四轮驱动”，转型振兴迈出了坚实步伐！我们本着对七台河发展和人民群众高度负责的态度，直面矛盾、勇于担当，改革创新、攻坚克难，干成了一些多年想干未干成的大事要事，破解了一些多年想解决未解决的难题。一些重点领域实现新变化、新突破，民营经济发展掀起新一轮热潮，全省纳税人满意度调查综合排名第一；在全省率先打响“第一炸”，取得淘汰煤炭行业落后产能攻坚战阶段性重大胜利，地方煤矿小散乱问题得到彻底根治；鼓励科技创新、扶持民营经济发展经验做法在全省、全国大型会议上作典型发言，构建新型“碳”产业加快转型发展获国务院通报表扬。

　　涓涓细流，终成大海，成绩来之不易。这是在省委、省政府和市委坚强领导下取得的结果，是在历届市委市政府打下的坚实基础上取得的结果，是在市人大、市政协监督支持下取得的结果，是全市人民团结奋斗的结果。在此，我代表市人民政府，向全市人民，向各位人大代表、政协委员，向各民主党派、工商联、无党派人士，向各人民团体、各界人士，向广大劳动者、企业家们，表示衷心的感谢和崇高的敬意！

　　各位代表！实现七台河转型发展、全面振兴全方位振兴，绝不是轻轻松松、敲锣打鼓就能实现的，我们决不能为取得的成绩而沾沾自喜，更不能有丝毫懈怠，必须风雨兼程、再接再厉。要清醒看到，习近平总书记指出的“四大短板”“三偏问题”在我市都不同程度存在。新兴产业发展不快、替代产业支撑力不强，淘汰煤炭落后产能短期影响不可忽视；生态环境修复治理任务艰巨，资源和环境约束趋紧；安全生产基础仍不牢固、社会治理还存在薄弱环节，教育、医疗、文化、养老等方面服务供给与人民群众期盼仍有差距，发展不平衡不充分问题仍比较突出；干部群众思想解放不够，营商环境需进一步优化，等等。这些问题，既有发展中客观规律性矛盾，也有我们思想观念、工作方法、工作作风上的问题。在今后的工作中我们必须付出更为艰苦的努力，苦干、实干、加油干，不忘初心，回应人民新期待，牢记使命，奋进时代新征程，不管乱云飞渡、风吹浪打，一步一个脚印把七台河转型振兴的伟大事业向前推进。

　　二、2019年重点工作

　　2019年是新中国成立70周年，是决胜全面建成小康社会的关键之年，是完成“十三五”规划目标任务承上启下之年，更是我们推进煤城转型发展，实现全面振兴全方位振兴攻坚克难、大有作为的一年。做好2019年政府工作的总体要求是：以习近平新时代中国特色社会主义思想为指导，全面贯彻落实党的十九大、十九届二中、三中全会和中央经济工作会议精神，聚焦贯彻落实习近平总书记在深入推进东北振兴座谈会上重要讲话和考察龙江时重要指示精神，深入贯彻落实省委十二届四次全会和市委九届三次全会精神，紧紧围绕统筹推进“五位一体”总体布局、协调推进“四个全面”战略布局，全面落实新发展理念和高质量发展要求，坚持稳中求进工作总基调，深入解放思想，激发内生动力，坚持民营富市、工业强市、生态立市、创新兴市战略，强化市场带动、要素推动、投资拉动、创新驱动，加快三产融合、虚实融合、产城融合、城乡融合，实施“五大振兴工程”“二十项利民行动”，统筹做好稳增长、促改革、调结构、惠民生、防风险各项工作，重塑投资营商新环境、重聚产业发展新动能、重构协调发展新格局，进一步巩固提升政治生态好、经济生态优、自然生态美的发展优势，奋力开创七台河转型发展，全面振兴全方位振兴新局面。综合考虑各方面情况，经济社会发展的主要目标是：地区生产总值增长4.5%左右，规模以上工业增加值增长4.0%左右，固定资产投资增长5.5%左右，一般公共预算收入按可比口径增长5.0%左右，城镇居民人均可支配收入增长5.5%左右，农村居民人均可支配收入增长6.0%左右，城镇登记失业率控制在4.5%以内；节能减排和大气、水环境质量确保完成省下达目标任务。

　　（一）实施产业振兴工程，推动经济高质量发展。加快转型发展，实现全面振兴全方位振兴，必须坚定发展才是硬道理，准确把握高质量发展内涵要求，坚持以项目建设为主要抓手，以产业转型升级为主攻方向，促进“六大产业”集群化、集约化发展，加速新旧动能接续转换。

　　加快产业结构优化升级。坚持走好绿色转型发展“三条根本出路”，实施《产业振兴三年发展规划》，完善“六基地、两中心”产业布局，加快构建多点支撑、多元发展、多业并举的现代产业体系。大力发展新型“碳”产业。深入贯彻“煤头化尾”，坚持循环化、园区化、高端化、多联产思路，推进隆鹏20万吨清洁化学品、泓泰兴18·30等项目投产达效，加快龙洋焦化二期及甲醇液氨联产、华夏一统复合肥、宝泰隆中间项炭微球、高软化点沥青、兴勃生物质复合肥、旭丰焦炉气综合利用等项目建设，推动煤化工产业向精细化工、合成化工、材料化工方向发展。做强石墨和石墨烯新材料产业，加快推进万锂泰负极材料、鑫科纳米石墨烯防腐涂料、中航铁路石墨烯新材料、暖丰公司石墨烯电暖材料、兴盛高纯石墨等项目建设。做大制造和再制造产业，加快双叶公司智能制造项目建设，支持勃农高速秸秆还田整地机、免耕播种机扩大产能批量生产，推动三江变压器与广东江门海鸿电气合作，开发新产品，拓展新市场。围绕再生资源集聚和综合利用，推动天鑫再生铅项目落地建设，亿海再生铝、裕盛源废轮胎裂解等项目开工生产。加快发展生物和医药产业，推进中瑯生物5 万吨衣康酸10 万吨葡萄糖酸钠及叶酸和清香型白酒、兴粮医药中间体、哈药总厂原料药等项目建设。积极发展商贸物流业，促进新零售小镇、商贸博览城二期、亚太国际商城等项目按期投入运营，构建智慧商圈。加快培育创意经济、共享经济等新业态，鼓励发展跨境电商、线上线下融合等新模式。出台降低物流成本十条硬措施，推进传化鑫达源物流、中石化东部区成品油仓储中心、三聚隆鹏物流园区等项目建设，完善物流配送集散体系，构建现代物流供应链、保障链。大力发展文化旅游产业，与周边地市建立旅游联盟，开发特色旅游产品，打造森林游、工业游、红色游、乡村游等精品旅游路线，加强重点景区的规划设计和策划包装，重点推进兰棒山抗联小镇、吉兴湖景区、高铁站花海公园等“十大景区”“十大展馆”建设。

　　大力推进产业项目建设。坚持把产业项目建设作为“一号工程”。在扩大项目投资规模上攻坚，全年重点推进5000万元以上产业项目50个，总投资270亿元，年度计划投资56亿元。主动对接国家和省重大战略，抓紧谋划一批大项目，力争进入全省“百大项目”集群。在提升项目质量效益上攻坚，结合资源禀赋和环保要求，谋划布局一批科技含量高、产出效益好、环境影响小的项目。坚持盘活“僵尸企业”和“腾笼换鸟”齐发力，不断提升土地产出率、资源利用率、效益贡献率。在加快项目推进速度上攻坚，成立市大项目办，推行项目建设“双向备案承诺制”，严格落实重点产业项目“四帮四保”制度，强化项目建设要素保障，在土地供给、环境评价、项目申报、银企对接、人力资源服务、法治安全保障等方面，指定专人、专班提供“保姆式”服务，切实提高投资完成率、新上项目开工率、续建项目投产率。

　　全方位开展招商引资。坚持把招商引资作为经济发展的生命线，建立“专班+园区”“目标+考核”的招商引资新机制，进一步加大招商引资力度。围绕“六大产业”集群发展靶向招商、精准招商，开展专业化招商、市场化招商和以企招商、以商招商、平台招商，力争在招大引强上取得新突破。抢抓长三角、珠三角、京津冀产业转移之机，加快七台河商会建设，延伸招商合作触角，抓好产业承接，提高项目落地成功率。大力支持域外企业在我市设立地区总部和研发中心、结算中心等功能性机构。坚持内引外联相结合，深入开展“七商回归”和“七智回归”活动，吸引七台河籍客商和人才回乡兴业创业。

　　提升园区平台承载能力。按照功能定位明晰、关联产业集聚、要素供给便利原则，统筹全市产业发展和空间布局，全面启动江河融合绿色智造产业园区建设，继续推进开发区及现代煤化工、石墨新材料、生物技术、再生资源、健康产业园等园区的水电路网、环保设施、孵化设施和公共服务设施建设，为产业集聚发展提供支撑和保障。加大园区开发和运营模式改革创新力度，探索推进园区管理企业化、运营市场化改革，推动园区发展质量变革、效率变革、动力变革，打造数个百亿级产业园区，形成转型振兴新的增长极。

　　（二）实施科技振兴工程，打造创新型城市。加快转型发展，实现全面振兴全方位振兴，必须坚持创新兴市战略，深刻把握科技对经济发展的引领推动作用，加快完善科技创新平台建设，引导科技创新人才集聚，加速科技成果转化，激发创新发展活力，全面提升产业发展层次和水平。

　　建设未来科技城。规划9.5平方公里“未来科技城”功能新区，打造城市副中心，挖掘新的动力源，培育新的增长极。谋划打造科技孵化区、科技金融区、科技文创区、科技总部区、科技教育区、科技休闲商贸区、科技场景试验区七大板块。重点推进清华双创小镇、同方科技园、人力资源产业园、青少年科技馆、国家石墨烯检测中心、产业技术创新中心、众创空间等科技创新载体，新零售小镇、生态智慧小镇、专家公寓、五星级酒店、科学家公园等配套服务载体建设，促进创新人才、创新要素和科技产业集聚发展，打造龙江东部科技创新研发高地。坚持整体推进、分步实施，2019年启动“月亮湾”3平方公里核心区规划建设，重点推进同方科技园等创新载体建设，完成开发区创业中心、宝泰隆创新大厦等重大设施和项目建设。

　　大力发展数字经济。紧跟大数据、人工智能、虚拟现实等科技变革步伐，加大互联网、物联网信息基础设施建设，推进4G 网络深度覆盖和5G 网络建设布局，加快联通公司“新型政务云”平台、华为和偶然大数据平台项目建设。推进数字经济与实体经济融合，鼓励机械、家具、食品、采矿等传统产业实施“两化融合”改造，建设智慧工厂、智慧矿山，优化生产、营销、管理各环节效率，推动企业降本增效。推进数字经济与城市管理、公共服务融合，大力推动科教、健康、环保、交通、旅游、安全等民生领域的“互联网+”应用，加快智慧城市建设，普及信息化服务。

　　强化人才技术引进。继续实施“双百”人才引进计划，力争在柔性引进两院院士、“长江学者”等高层次人才方面取得更大进展。实施“头雁行动”，引进培养领军人才，给予平台、资金、政策、环境支持，打造“拴心留人”好环境。坚持政府主导，推动政产学研用深度融合，引导企业与高校及科研机构开展深度合作，促进创新链和产业链“双向融合”。发挥北京石墨烯研究院宝泰隆研发中心、鑫科纳米院士工作站、省工研院七台河分院、黑大工研院七台河分院等机构的科技研发优势，加快高新技术成果转化。重点推进碳转化合成汽油、钛电池、快淬钕铁硼磁体等成果转化落地。

　　加快培育创新主体。实施新一轮科技型企业三年行动计划，培育一批技术含量高、有发展前景的科技型企业。发挥企业创新主体作用，持续推进产品创新、技术创新、科技创新，从跟跑到领跑，争创更多全国全省第一、唯一。积极引导企业通过购买专利或技术合作等方式，加强创新技术应用和推广，提升企业创新发展能力。完善政府科技创新投入保障机制，安排总额不低于1000万元/年的专项经费，支持应用技术研究与开发；设立1000万元科研担保金、1000万元重大科技专项配套资金，降低科技成果转化风险；连续三年安排1000万元专项经费支持北京石墨烯研究院宝泰隆研发中心石墨烯新材料成果研发，投入1000万元专项经费支持北京紫顶光合碳转化合成汽油项目。

　　（三）实施乡村振兴工程，加快农业农村现代化。加快转型发展，实现全面振兴全方位振兴，必须坚持农业农村优先发展，以深化农业供给侧结构性改革为主线，按照产业兴旺、生态宜居、乡风文明、治理有效、生活富裕的总要求，推进实施乡村振兴战略规划，促进农村一二三产业融合发展，建设美丽乡村。

　　推进农业供给侧结构性改革。加快种植业结构优化调整，推动种植业向绿色粮仓、绿色菜园、绿色厨房转型发展。发展优质高效粮食生产，确保粮食安全。扩大高淀粉玉米、高蛋白大豆、高钙富硒水稻、有机谷子、鲜食玉米等特色作物种植面积，因地制宜发展食用菌、山野菜、北药、坚果、浆果等林下经济作物，增加高端绿色食品供给。推进养殖业向特色化、标准化、规模化发展，大力发展野猪、梅花鹿、肉驴等特色养殖，加快天兆百万头生猪项目建设。做好非洲猪瘟等重大动物疫情监控、防控工作。抓好“互联网+农业”示范基地创建，促进节水灌溉、可视农业、现代农机等技术普及应用，围绕促进农业生产，加速构建大中小微结合、骨干和田间衔接的农村水利基础设施网络。打好寒地黑土、非转基因、绿色有机优势牌，不断扩大“三品一标”认证规模，完善农产品质量追溯体系。发展壮大家庭农场、种养殖大户、合作社等新型农业经营主体，鼓励发展农民专业合作社联合社，促进小农户与现代农业有机衔接。

　　推进农村一二三产融合发展。深入发掘农业农村的生态涵养、休闲观光、文化体验、健康养老等功能价值，加强农工商互联，密切产加销衔接。深入推进“两头两尾”，大力发展水稻、玉米、大豆等精深加工产业，提高农产品加工转化率，延长产业链、提升价值链，重点推进圣海蓝靛果深加工、宏泰松果松仁深加工、润池科技食用菌、景天农业科技园等项目。推动设施农业、休闲观光农业发展，加快龙谷田园综合体、田润农业生态园、金沙乡村度假综合体等项目建设。推广“订单收购+分红”“土地流转+优先雇用+社会保障”“农民入股+保底收益+按股分红”等多种利益联结方式，让农民分享加工、销售环节收益。谋划建设浆果坚果等全国性特色农林产品集散批发市场，加大绿特色农产品在电商、微商平台线上销售力度，拓宽渠道，创建品牌，推广错时销售、定制销售，实现“种得好，卖得更好”。

　　推进农村人居环境整治。编制实施农村布局和农村建设两个规划。建设以乡镇政府驻地为中心的农村经济圈，合理配置公共服务设施，构建便捷的生活圈、完善的服务圈、繁荣的商业圈。加快通村道路硬化建设，在“村村通”基础上，努力实现“屯屯通”。实施农村电网改造升级，提高供电可靠率，实现光纤宽带网络和移动通信网络乡村全覆盖。持续开展农村人居环境整治三年行动，推进“厕所革命”“垃圾革命”“污水革命”，实施农村“三清四改一绿化”，加强村屯垃圾存放和收转设施建设，加快粪污、畜禽养殖废弃物无害化、资源化利用，年底前农村生活垃圾无害化处理率达到50%以上。推进“大棚房”、公共空间和庭院环境整治。深入挖掘乡村特色文化符号，加大宏伟镇等特色小镇、四新村等乡村旅游示范村建设，重现田园风光留住黑土乡愁。

　　（四）实施生态振兴工程，打造宜居宜业美丽家园。加快转型发展，实现全面振兴全方位振兴，必须坚持以习近平生态文明思想为指导，统筹推进生态文明建设，坚决打好污染防治攻坚战，建设美丽宜居七台河。

　　坚决打好蓝天保卫战。全面加强企业污染防治，编制大气污染源排放清单，实施工业污染源全面达标排放计划，加快焦化、火电、涉煤、水泥等重点行业污染超低排放改造。强化扬尘面源污染治理，强力推进施工扬尘、道路扬尘、车辆扬尘管控，洗煤、焦化、建材等重点企业物料堆场全面实施封闭运行。继续开展建成区10蒸吨及以下燃煤小锅炉“清零”行动。加大餐饮业及露天烧烤油烟治理力度，坚决管住群众身边的空气污染。强化秸秆禁烧，促进“五化”综合利用，秸秆综合利用率提高到65%以上。

　　坚决打好碧水保卫战。严格落实河湖长制，实施河流和湖库分流域、分单元、分阶段一体化治理，集中开展河湖“清四乱”专项行动，持续改善水系环境质量。加强桃山湖生态水体保护，抓好倭肯河流域综合治理，严控沿岸企业污染排放，继续推进万宝河、新老七台河、碾子河等黑臭水体治理，力争2019年底基本消除黑臭水体。强化水源地保护，加快汪清水库水源地保护区划定和规范化建设，整治城市水源保护区内违法违规问题。打好地下水管理和保护战役，深化地下水污染防治。2020年底前，城市和县城污水处理率分别达95%和85%。

　　坚决打好净土保卫战。开展土壤污染状况详查，实施土壤污染管控修复，分类别、分用途、分阶段治理，将建设用地土壤环境管理要求纳入用地规划和供地管理。实施城镇垃圾治理能力提升三年行动计划，完善餐厨垃圾、生活垃圾、医用垃圾等分类配套体系，加强末端收集系统建设，推进垃圾处置无害化、减量化、资源化。加强黑土保护，实施农业“三减”行动，加强农用地土壤环境分类管护，严控新增污染、减少存量污染，保障农产品质量和人居环境安全。

　　坚决打好生态保卫战。实行一条红线管控生态空间，统筹山水林田湖草综合治理，提升生态系统质量和稳定性。全面推行生态保护补偿机制，落实企业修复治理主体责任，推动煤炭、化工、农副产品加工等传统产业清洁化改造，建设绿色矿区、绿色工厂、绿色园区。大力发展环保产业，探索合同能源管理、节水管理、环境污染第三方治理等节能环保服务新业态。按照“宜林则林、宜耕则耕、宜草则草、宜景则景”原则，加快矸石山、棚改腾空土地治理和综合利用。实施天然林储备工程，发展“碳汇”经济。严肃查处违规挤占生态空间、破坏自然遗迹等行为，坚决守好森林、湿地、湖库、冰雪等资源。

　　（五）实施改革振兴工程，释放经济发展活力。加快转型发展，实现全面振兴全方位振兴，必须高举改革开放旗帜，坚持“五个有利于”，深化重点领域改革，坚决破除制约发展的体制机制弊端，不断注入新动力、增添新活力、拓展新空间。

　　推动民营经济快发展大发展。实施“百千万”提升工程，推动规模上企业转型升级、中小微企业提质增效、个体户发展壮大。坚持非禁即入，拓宽民营经济发展空间。落实完善“黄金十条”系列扶持政策，推出一系列含金量更大的“政策包”，增强政策扶持的针对性、实效性。健全资源要素保障机制，持续推进减费降税，最大限度降低企业生产经营成本。鼓励民营企业引进新技术、应用新工艺、开发新产品，加快转型升级，造就一批主业突出、竞争力强、纳税超亿元实力民企。高度重视提升民营企业家素质，大力弘扬企业家精神，壮大企业家队伍，释放企业家才能。继续开展“企业家大讲堂”“企业家进高校”活动，每年安排100 名民营企业家到高校和国内外发达地区学习培训，拓展企业家的眼界和格局，增强战略思维和创新理念，提高经营能力、管理水平，完善法人治理结构。扶持小微企业健康成长，引导小微企业与大企业协作配套，立足“小、精、专、特”优势提质增效，加快“个转企”“小升规”。充分发挥“创客街区”等“一区十园”孵化平台作用，加大创业扶持力度，为小微企业成长壮大创造良好条件。

　　深化重点领域改革。深入贯彻落实“巩固、增强、提升、畅通”八字方针，深化供给侧结构性改革，坚决打赢淘汰煤炭落后产能攻坚战，加快保留矿井提能改造和新建矿井审批，建设一批规模化、标准化、智能化矿井。深化国企国资改革，试行市属国有企业总经理公开招聘和中层干部竞聘上岗制度，推进公交、公立医院等集团化改革。支持七矿公司“三项制度”改革，加快辅业单位改制。推进矿区转供电管理体制改革。深化农业综合改革，加快农村土地“三权分置”改革，完成农村集体产权制度改革，健全农业新型社会化服务体系。深化事业单位机构改革，全面精简压缩事业单位编制规模和数量，确保完成两个精简10%任务。深化财政评审、政府采购制度改革，提高财政评审、政府采购效率。按时完成事业单位和国有企业车改目标任务。加大政府购买服务力度，加速推进环卫、公务用车、机关事务服务等公共服务领域市场化进程。深化金融体制改革，加快农信社股份制改造，成立七台河市农商行。强化对金融机构存贷比、加权平均贷款利率等数据综合评价考核，提升金融支持实体经济效率。做好龙煤、农垦、森工改革承接工作。

　　持续扩大对外开放。加强对外交通体系建设，加快七宝铁路、七密公路、通用机场、航空小镇谋划推进，积极对接同江铁路口岸，打通陆海联运国际贸易通道。全力办好首届丝路电商国际合作会议，继续举办第三届石墨烯应用创新创业大赛、七台河·江门招商推介会等交流活动，积极组织参加“进博会”“哈洽会”“绿博会”“中俄博览会”等大型会展经贸活动，进一步拓展城市“朋友圈”提升影响力。深入推进“江河”合作，加快两市合作框架协议落实，加大在园区共建、产业互补、科技创新、康养旅游、人文交流等领域合作，积极参与江门农博会、花样嘉年华等大型展会论坛，利用江门全球华侨联络站优势，积极引入大湾区及国际资本。主动融入“一带一路”，借助俄罗斯远东开发有利时机，在矿产资源开发、农产品基地建设和再生资源回收利用等领域开展对俄合作。利用新零售小镇保税国际平台优势，鼓励企业开展跨境电商进出口业务，支持建设木制品、农副产品、煤化工产品进出口基地，力争全年对外贸易备案企业突破100 家。

　　深度优化营商环境。深化“放管服”改革，依托市民中心建立多级互联数据共享平台，全面推行政务服务“网上办、一次办、我帮办”，落实“四零服务”承诺制，推进审批流程再造，实行并联审批、多评合一，大幅压缩企业施工许可、生产要素供给审批报装时限。继续深化商事制度改革，拓展“多证合一”范围，企业开办时间压缩至5个工作日。开展“证照分离”改革，完善“证后”监管机制，提高监管效能。持续清理涉企收费，落实增值税降税率、留抵税额退税政策，提高小规模纳税人年销售额标准，扩展减半征收所得税的小微企业范围。推动大用户直购电，降低企业用电成本。加强政银企对接机制建设，发挥政府担保机构、贷款周转金作用，鼓励和支持金融机构创新金融产品，加大对企业信贷投放力度。支持企业以债权、股权、基金等方式进入资本市场筹资融资，鼓励企业上市融资，加快鹿山集团上市步伐。优化政商环境，建立营商环境评价机制，整治不作为、乱作为等影响发展环境突出问题。落实“企业宁静日”制度，实施“双随机一公开”监管，推进综合执法体制改革，解决多头执法问题。严厉打击涉企犯罪行为，保障企业合法经营，打造公平正义的法治化营商环境。落实企业家“零距离”沟通协调机制，发挥“微市长”“微区长”“微局长”沟通平台作用，开办“市长茶馆”，整合“市长热线”，坚持市长早餐会、双月恳谈会、季度问诊会、年度联谊会制度，主动向企业问政问计问需，及时解决企业发展中的问题，提供精准服务，全面营造亲商、重商环境。

　　（六）实施20项利民行动，全面提升百姓幸福指数。加快转型发展，实现全面振兴全方位振兴，必须坚持以人民为中心的发展思想，持续加大民生投入，补齐民生短板，夯实底线民生，保障基本民生，提升品质民生，推动解困型民生向幸福型民生转变，进一步增强人民群众的获得感、幸福感和安全感。

　　1、教育优先行动。全面落实教育优先发展战略，推进教育城乡一体化。新建九年一贯制学校，办成省内知名高校附属学校，打造区域名校。加大教育教学设施投入力度，完成市、区20所学校操场硬化改造、33所学校塑胶运动场地、13所学校旱厕改水厕工程；推进茄子河双鹤学校、新兴区第一小学、新建小学等学校建设，着力解决中小学“城挤乡弱”“大班额”等问题。改扩建公办幼儿园7 所，提升幼儿园普惠率。实施教育信息化工程，加快“三通两平台”建设，促进优质教育资源共享。高质量办好“三点半课堂”，减轻学生、家长负担。加强儿童青少年近视防控工作，抓好短道速滑特色学校建设，切实提升学生体质健康水平。适应新高考模式，深化教育领域综合改革，建立校长、教师定期轮岗交流和定期外出培训制度，进一步推进教师绩效工资改革试点，加大优秀师资引进力度，提高教育教学水平。加快职业技师学院创业大厦、实验实训楼、公共实训基地建设，推进与黑龙江大学、江门职业技术学院对接，加强专业建设，与企业联合开展订单定向培训，扩大技工、技师等实用型人才培养规模。

　　2、医疗提质行动。推进“健康七台河”建设，深化公立医院综合改革和分级诊疗试点，扩大补充医疗保险试点，最大限度让群众少花钱看好病。继续加大与国内知名医院的合作力度，打造重点特色学科，全面推开农村远程医疗，提高城乡整体医疗技术水平。进一步落实“百名医科研究生计划”，加大全科医生的引进和培养力度，培育学科专家。推进人民医院医师培训基地、七煤医院综合楼、中医院康复中心和治未病中心等医疗基础设施建设项目如期投入使用。

　　3、“舌尖安全”保卫行动。加强对食品生产、流通、餐饮全链条监管，进一步清理规范转基因食品经营行为，严禁中小学、幼儿园食堂采购转基因食品。加快推进“阳光餐饮工程建设”，实现餐饮企业厨房可视化。完善食品药品检验检测体系，加强食用农产品、食品检验检测。加大对有毒有害食品添加剂和虚假药品的打击力度，规范药品宣传和销售。

　　4、就业促进行动。落实“稳就业”要求，推动更高质量和更充分就业。加快“电子就业地图”建设，抓好高校毕业生、转岗职工、农村转移劳动力、退役军人等群体就业技能培训和促进就业工作。继续开展“创业促就业”专项行动，推进就业创业三年行动计划，设立创业专项扶持资金，将就业专项资金使用由保障型向促进型转变，推动创业带动就业。

　　5、社保提档行动。全面推进城乡居民社会保障体系建设，完成全民参保计划，提高统筹层次、基金支撑能力和待遇水平，完善经办管理服务体系，努力实现社会保障法定人员全覆盖。做好七矿公司社保移交承接工作，推进市矿医保统筹，解决“一市两制”问题。完善统一的城乡居民基本医疗保险制度和大病保险制度，继续深化医保支付方式改革，全面落实总额控制和按病种付费制度。

　　6、脱贫巩固行动。坚决打赢脱贫攻坚战，巩固贫困县摘帽、贫困村出列、贫困人口脱贫成果，抓好驻村帮扶工作，因村因户因人施策，精准落实各项扶贫政策。加强动态管理，对脱贫户进行“回头看”，对“边缘户”进行重点扶持，保持扶贫政策的连续性。推进扶贫产业加快发展，保证贫困户持续稳定增收，加强光伏扶贫电站运营管理，完善收益分配机制。建立扶贫基金，对因病因残致贫人口进行救助，助力脱贫、防止返贫，决不让一名贫困群众掉队。

　　7、“夕阳”关爱行动。不断满足老年人持续增长的养老服务需求，加快构建居家为基础、社区为依托、机构为补充、医养相结合的养老服务体系，为老年人提供精准化、个性化、专业化服务。鼓励社会力量兴办养老服务机构，加快养老服务业和“候鸟养老”产业发展，加快推进天赐智慧医养中心项目建设，谋划建设春晖养护院二期项目。

　　8、扶危济困行动。统筹社会救助体系建设，完善最低生活保障制度。加快推进未成年人保护中心、红十字会应急救援基地项目建设。加大对残疾人、失独老人、孤儿、农村留守老人和儿童、流浪乞讨人员等弱势群体帮扶救助力度，提升社会弱势群体生活保障水平。

　　9、全民健康行动。落实“健康中国2030”规划纲要，倡导健康生活方式，广泛开展“三减三健”行动，提升城乡居民在科学健康观、传染病防治、慢性病防治、安全急救等方面素养，持续提高人民健康水平。积极开展“助力2022冬奥会”“百万青少年上冰雪”等全民健身活动，营造全民运动、全民健身的浓厚氛围。巩固扩大短道速滑优势，做好冬季项目跨界跨项选材等体育后备人才培养工作，不断提升我市竞技体育竞争力。

　　10、文化惠民行动。加强公共文化设施建设，完善市文化中心配套功能，谋划建设肃慎文化馆、文化艺术馆、许镜清音乐馆。提高图书馆、博物馆、群众艺术馆、短道速滑冠军馆免费开放质量，开展系列主题文化活动，把中俄青少年短道速滑国际邀请赛、彩灯游园等“冰雪嘉年华”活动打造成品牌，挖掘提炼城市历史文化资源，打造《冰之梦》《兰棒山抗联小英雄》等精品力作，满足群众文化需求。做好文物保护、文化遗址发掘申报、非物质文化遗产保护与传承工作。深入实施城乡文明行动三年计划，持续开展“引领风尚、铸魂煤城”系列活动，为转型振兴发展提供强大精神力量。

　　11、路网畅通行动。坚持着眼长远，统筹布局，规划先行，以路为纲，拉开城市骨架。加快推进牡佳客运专线七台河段及客运综合枢纽工程建设。打通半截路，治理拥堵路，新建、改造、扩建大同、宝安、庆余、环湖、同仁、山湖、经一、文华、奋斗、风采、七桦等16条街路，改造景丰路与大同街、杨扬大街与308省道、七依路与七勃路三处道路交叉口，形成快速畅通城市路网。

　　12、安居保障改善行动。加强保障性住房建设，努力满足群众安居需要。加快西部矿区老旧危楼、东胜村、自建区等城镇棚户区居民搬迁安置，新建3000套、货币化安置7977套。健全中低收入家庭住房信息管理，新增公共租赁住房租金补贴保障对象1000户。完成四个片区的老旧小区综合改造工作。继续推进农村危房（泥草房）改造，改善农村居住条件。

　　13、公共服务提效行动。加快供水管网二期改造、水厂升级、水质监测中心、智慧水务、汪清水库备用水源管线等项目建设，保障供水安全和水质安全，实现城区24 小时供水全覆盖。继续推进市区老旧供热管网改造，启动地温能供热工程。实施文华路北燃公司整体搬迁，积极推进俄气入境和新型清洁能源置换前期准备工作。开工二污4万吨/日扩建工程，完成一污2万吨/日扩建和市区4.2公里污水管网改造，新建城乡结合部污水管网50公里，启动开发区、江河园区、宏伟镇污水处理厂建设。推动危废处置中心项目建设，完成垃圾处理场防渗工程。

　　14、园林城市提标行动。大力开展城市绿化美化，全面推进桃山湖湿地公园建设，实施主要街路及中心区节点景观建设。贯彻实施城市公园条例，持续推进仙洞山公园、人民公园、应龙山公园、万宝公园改造升级，精品化建设口袋公园，新增绿化面积200万平方米，建设仙洞山市民休憩中心及北侧人行木栈道、环湖路亲水木栈道、月亮湾栈桥。加大佳境街等路灯盲点街路亮化建设，实施文华路沿街楼体亮化，升级警官医院、新华书店等楼体亮化，在主要街路和重要节点打造城市“灯光秀”，巩固提升国家园林城市品位。

　　15、市容环境治理行动。开展城市管理提升年行动，加大对违法建筑、占道经营、农贸市场整治力度，实施七彩城道路和背街小巷环境整治。推进环卫市场化，提高环卫“四清”机械化作业率，实施精细化保洁。落实物业管理社会评价及末位淘汰制度，提升物业服务水平。坚持“六化一考”，落实“门前五包”，拿出“绣花功夫”加强城市精细化管理，推动智慧城市管理平台建设。

　　16、安全生产整治行动。牢固树立安全发展理念，严格落实安全生产责任制，切实做到“凡事有人负责、凡事有章可循、凡事有据可查、凡事有人监督”。强化制防、人防、技防综合监管措施，加强煤矿“内功、外功、基本功”建设，打牢煤矿安全生产基础。持续开展危险化学品、道路交通、建筑工地、消防森防、特种设备、城镇燃气、人员密集场所等行业领域安全生产大检查和专项整治，坚决防范和遏制安全事故发生。推进七台河综合应急救援基地建设，完善和落实突发事件应急联动机制，不断提升安全保障能力。

　　17、社会治安防控行动。坚持公安与武警联勤武装巡逻，强化重点区域、重点部位的管控力度，确保“白天见警察、晚上见警灯”，最大限度挤压违法犯罪空间。深入推进“雪亮工程”建设，加强立体化、信息化社会治安防控体系建设，提升社会治安动态防控能力。依法打击各种违法犯罪行为，深入推进扫黑除恶专项斗争，不断提高人民群众安全感和满意度。高标准建设全国反洗钱领域骨干联勤中心，深入开展“9·8”专案攻坚，有效防范金融风险。

　　18、禁毒城市创建行动。加快推进禁毒工作社会化进程，构建齐抓共管、全民参与禁毒工作格局。充分发挥禁毒教育基地作用，开展“七进”等宣传教育活动，营造全民禁毒氛围。建立健全毒情研判和多警种联动打击涉毒违法犯罪工作机制，持续开展“两打两控”专项行动，形成打击毒品犯罪高压态势。深入实施社区戒毒康复“831”工程，落实“3+N”社会帮教，帮扶吸毒人员回归社会。

　　19、社区治理提升行动。推动社会治理重心向基层下移，加强城乡社区服务设施建设，突出解决好社区活动用房不足问题。健全完善网格化社会治理机制，发挥居民自治组织和社会组织在社会治理中的重要作用，有序推进基层自治，实现政府治理和社会调节、居民自治良性互动。加强社区工作者队伍专业化建设，发挥好社区服务中心窗口便民作用，为社区居民提供精准有效的公共服务和公共管理。

　　20、矛盾调处化解行动。坚持发展新时代“枫桥经验”，发挥人民调解、行政调解、司法调解联动作用，健全落实社会矛盾源头预防、排查预警、多元化解机制。强化公共法律服务体系建设，提供便捷化、精准化公共法律服务。推进群众诉求服务中心标准化、规范化建设，加快“12345”热线整合，提升信访服务能力水平。严格落实信访工作责任制，集中开展进京越级访专项治理，围绕征地拆迁、劳资纠纷、环境污染等重点领域，开展专项攻坚行动，依法及时就地解决群众合理诉求，维护社会和谐稳定。

　　三、加强政府自身建设

　　加快转型发展，实现全面振兴全方位振兴是新时代党和人民赋予我们的神圣使命，必须坚持以习近平新时代中国特色社会主义思想为引领，提高政治站位，强化使命担当，深入解放思想，转变工作作风，增强凝聚力、激发创造力、强化执行力、提高战斗力，着力打造群众最满意、企业最满意的“两最”政府。

　　强化理论武装，建设学习型政府。深入学习贯彻党的十九大精神，全面贯彻落实习近平总书记在深入推进东北振兴座谈会上重要讲话和在我省考察时重要指示精神，树牢“四个意识”，坚定“四个自信”，坚决做到“两个维护”，坚定不移贯彻落实党中央国务院、省委省政府和市委各项决策部署。坚持学用结合，更新知识储备，打牢全面、系统、专业的知识根底，提高适应新时代、实现新目标、落实新部署的专业化能力。

　　深入解放思想，建设创新型政府。坚持在提升行政效能上解放思想，发扬“敢”字精神，抢抓发展机遇，强化顶层设计，积极推进政府治理理念创新、服务模式创新、体制机制创新。开展“学江门、找差距、谋转型、促振兴”学习年活动，学习江门在创新精神、服务意识、法治思维、市场化理念等方面的做法经验，对标找差、取长补短，切实把解放思想转化为促进高质量发展的动力源泉。对标中央、省机构改革要求，调机构、优职能，实现政府组织机构和人员编制科学化、规范化、法制化。

　　深化作风整顿，建设实干型政府。围绕创优发展环境，驰而不息纠“四风”转作风，下决心下气力集中整治形式主义、官僚主义突出问题，进一步提升政府服务效能。深化政府绩效管理，强化目标任务考核，铆紧各方责任，狠抓工作落实，持续提升政府执行力。加强和改进调查研究，坚持问题导向、目标导向、需求导向、公平导向、效率导向，找准工作发力点，做到心中有底、心中有数、心中有谱。强化“一切围着企业转，一切围着项目干”的“店小二”服务理念，增强前瞻思维、专业素养、务实干劲，打造“胸有全局、心有规矩、肩有担当、业有专攻”干部队伍。

　　坚持依法行政，建设法治型政府。牢固树立法治观念，把法治精神、法治思维、法治方式融入政府工作全过程，严格依法履职、依法用权、依法办事。严格执行“公众参与、专家论证、风险评估、合法性审查、集体讨论决定”重大行政决策程序，发挥法律顾问、新型智库作用，提高依法决策、科学决策、民主决策水平。自觉接受人大法律监督、政协民主监督，办理好人大代表建议和政协委员提案。全面推进政务公开，自觉接受全社会监督。

　　严守纪律规矩，建设清廉型政府。贯彻落实全面从严治党各项要求，严守党的纪律和规矩，不断加强政府系统党风廉政建设和反腐败斗争。强化源头治理，加强审计和巡察结果运用，严管公共资金，严管公共资源交易，严管国有资产资本，严格控制“ 三公”经费和一般性支出，树立让百姓过“好日子”政府必须过“紧日子”思想，把有限的资源和财力用在推动发展、改善民生上，始终保持人民政府的清廉有为形象。

　　各位代表！实现七台河转型发展全面振兴全方位振兴，是全市人民的共同期盼，需要使命担当，呼唤拼搏实干。没有等来的辉煌，只有拼出的精彩。让我们更加紧密地团结在以习近平同志为核心的党中央周围，高举习近平新时代中国特色社会主义思想伟大旗帜，在市委的坚强领导下，在市人大和市政协监督支持下，解放思想、迎难而上，大力发扬“敢为人先，勇争一流”的七台河精神，坚定小城市有大作为的信心，笃定小城市有大发展的决心，做好转型“答卷”，走好振兴“长征”，为建设“精美富强”现代化新七台河，打造幸福之城而努力奋斗！以优异成绩庆祝中华人民共和国成立70周年！
